# T2 Values of Posterior Horns of Knee Menisci in Asymptomatic Subjects

**DOI:** 10.1371/journal.pone.0059769

**Published:** 2013-03-28

**Authors:** Shih-Wei Chiang, Ping-Huei Tsai, Yue-Cune Chang, Chao-Ying Wang, Hsiao-Wen Chung, Herng-Sheng Lee, Ming-Chung Chou, Yi-Chih Hsu, Guo-Shu Huang

**Affiliations:** 1 Graduate Institute of Biomedical Electronics and Bioinformatics, National Taiwan University, Taipei, Taiwan, ROC; 2 Department of Radiology, Tri-Service General Hospital, National Defense Medical Center, Taipei, Taiwan, ROC; 3 Imaging Research Center, Taipei Medical University, Taipei, Taiwan, ROC; 4 Department of Radiology, Wan Fang Hospital, Taipei, Taiwan, ROC; 5 Department of Mathematics, Tamkang University, Taipei, Taiwan, ROC; 6 Department of Biology and Anatomy, National Defense Medical Center, Taipei, Taiwan, ROC; 7 Department of Pathology, Tri-Service General Hospital, National Defense Medical Center, Taipei, Taiwan, ROC; 8 Department of Medical Imaging and Radiological Sciences, Kaohsiung Medical University, Kaohsiung, Taiwan, ROC; National Yang-Ming University, Taiwan

## Abstract

**Purpose:**

The magnetic resonance (MR) T2 value of cartilage is a reliable indicator of tissue properties and therefore may be used as an objective diagnostic tool in early meniscal degeneration. The purpose of this study was to investigate age, gender, location, and zonal differences in MR T2 value of the posterior horns of knee menisci in asymptomatic subjects.

**Methods:**

Sixty asymptomatic volunteers (30 men and 30 women) were enrolled and divided into three different age groups: 20–34, 35–49 and 50–70 years. The inclusion criteria were BMI<30 kg/cm^2^_,_ normalized Western Ontario and McMaster Universities (WOMAC) pain score of zero, and no evidence of meniscal and ligamentous abnormalities on routine knee MR imaging. The T2 values were measured on images acquired with a T2-weighted fat-suppressed turbo spin-echo sequence at 3T.

**Results:**

The mean T2 values in both medial and lateral menisci for the 20–34, 35–49, and 50–70 age groups were 9.94 msec±0.94, 10.73 msec±1.55, and 12.36 msec±2.27, respectively, for women and 9.17 msec±0.74, 9.64 msec±0.67, and 10.95 msec±1.33, respectively, for men. The T2 values were significantly higher in the 50–70 age group than the 20–34 age group (*P*<0.001) and in women than in men (*P* = 0.001, 0.004, and 0.049 for each respective age group). T2 values were significantly higher in medial menisci than in lateral menisci only in women age 50–70 (3.33 msec, *P* = 0.006) and in the white zone and red/white zone of the 50–70 and 35–49 age groups than that of the 20–34 age group (2.47, 1.02; 2.77, 1.16 msec, respectively, all *P*<0.01).

**Conclusion:**

The MR T2 values of the posterior meniscal horns increase with increasing age in women and are higher in women than in men. The age-related rise of T2 values appears to be more severe in medial menisci than in lateral menisci. Differences exist in the white zone and red/white zone.

## Introduction

The important role of the meniscus in knee osteoarthritis (OA) has been demonstrated in a few reports, where meniscal pathologies, such as meniscal degeneration and meniscal tear, are thought to be associated with changes in articular cartilage during the early or late stages of OA [1]–[3]. Modalities such as magnetic resonance (MR) imaging, recently used to examine the effects of progressive OA and to detect early structural and biochemical changes in the knee osteoarthritis cartilage, are imperative for effective diagnosis and treatment of OA [4], [5]. Specifically, MR T2 values have been shown to provide important information about changes in the collagen fiber network and water content of articular cartilage [6]–[10]. Several MR imaging sequences (including T2, T1rho [T1 relaxation time in a rotating frame], and dGEMRIC [delayed gadolinium-enhanced MR imaging of cartilage]) have also been developed to study the meniscus matrix [10]–[13]. Prior studies have indicated that T1rho has a dynamic range and is a highly sensitive detector of chondral change. Higher collagen concentration and lower proteoglycan content in the meniscus may account for the stronger relationship between T2 and chondral change in the meniscus [11], [12]. Several reports have validated the use of dGEMRIC (a proteoglycan-based imaging technique) [4], [10]. Its sensitivity to highly charged glycosaminoglycan (GAG) molecules makes it particularly useful for assessment of the biochemical properties of articular cartilage [4]. The MR T2 value is a reliable indicator of tissue properties, and therefore it may be used as an objective diagnostic tool in early meniscal degeneration. Thus, MR T2 value was a more suitable measure of dynamic change in the meniscus especially in our asymptomatic subjects. Studies have suggested that meniscal T2 behavior reflects OA-related micro-structural change [10], [12].

The meniscus is not a homogeneous tissue, and inner and outer areas of the meniscus differ in composition and cell metabolism [14], [15]. T2 values were shown to differ significantly between zones in the posterior horn menisci of healthy young subjects [16]. Therefore, regional differences may influence the diagnostic value of quantitative T2 measurements in menisci. In addition, aging and gender (both noted to have a significant effect on proteoglycan synthesis in human menisci [17], [18]) may also be factors that could affect the diagnostic value of quantitative meniscal T2 measurements. Thus, regional MR T2 behavior in the knee menisci should be established as a function of age and gender before meniscal T2 is used as a diagnostic index of early OA-related biochemical change.

The aim of this study was to determine the age, gender, location (medial vs. lateral) and zonal differences in MR T2 values of knee menisci in asymptomatic subjects. To our knowledge, relatively few studies have investigated such relationships in knee menisci using the T2 mapping method in a quantitative manner [11]–[13], [16]. Since the posterior horn of the medial meniscus is the most frequent location of meniscal degeneration and tears in primary OA of the knee [19], [20], the posterior horns of the menisci were selected as the focus of this study.

## Materials and Methods

### Ethics Statement

Each participant provided written, informed consent. The consent form and this study protocol were approved by the National Defense Medical Center and Tri-Service General Hospital institutional review board. The study was conducted in accordance with the Declaration of Helsinki.

### Subjects

**Figure 1:**
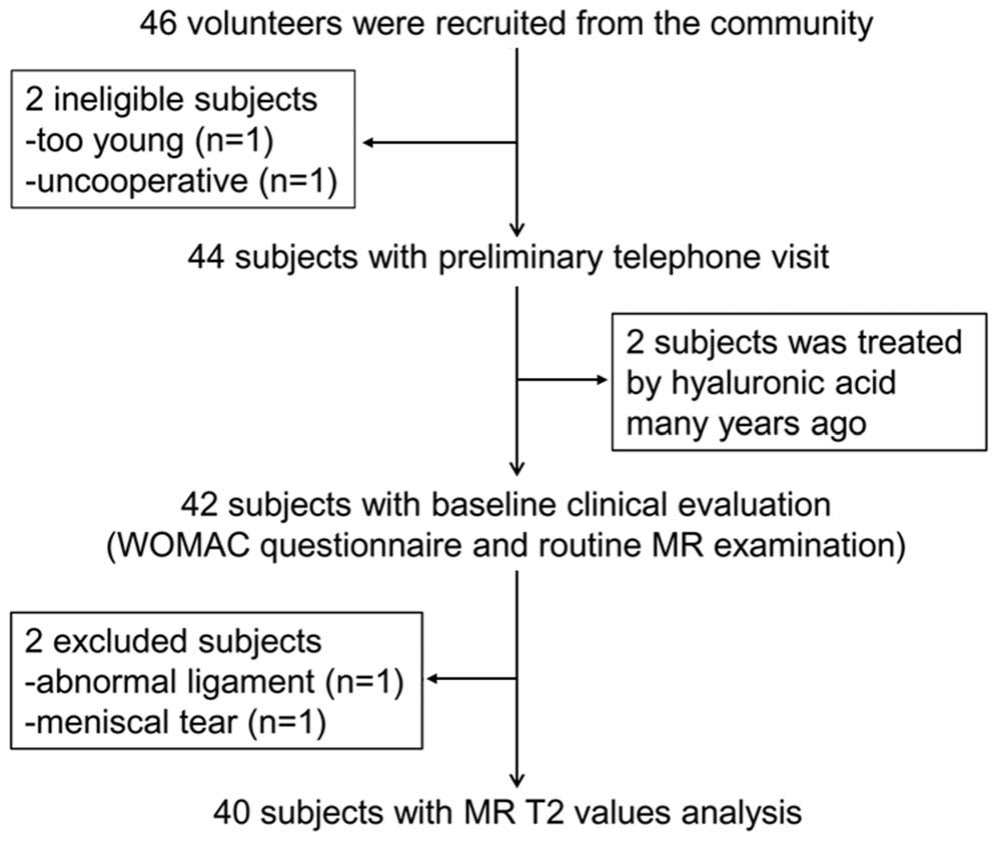
Flowchart shows the process used to select and recruit subjects from the local community.

Sixty asymptomatic healthy young volunteers (30 men and 30 women) were enrolled. Twenty subjects were from a prior study (with the same inclusion criteria as below) that measured MR T2 values of knee menisci, and 46 subjects were volunteers recruited subsequently [16]. All subjects were Taiwanese and recruited from the local community by way of print media. Inclusion criteria for the enrolled subjects were: (1) BMI <30 kg/cm^2^ [21], (2) asymptomatic with normal Western Ontario and McMaster Universities (WOMAC) pain score [22] of zero for both knees for more than 30 days before the baseline clinical visit, (3) no evidence of meniscal tear or meniscal intrasubstance fluid on MR imaging, (4) no evidence of ligamentous abnormalities on MR imaging, and (5) no evidence of an absence of any portion of the meniscus or a discoid meniscus. A meniscal tear was defined as an abnormal linear or complex high signal intensity communicating with the articular surface [23]. Exclusion criteria were age younger than 20 years, presence of OA risk factors, history of knee injury, obesity, high intensity exercise or sports, and loss of knee stability, history of non-OA knee arthropathy or chronic disease, or long-term medication or nutritional supplements use. As shown in the subject selection schema (Figure 1), 46 subjects underwent body height/mass measurement, completed the WOMAC OA questionnaire, and underwent routine MR examination before undergoing the T2 values examination. Six volunteers failed to meet the criteria mentioned above and were excluded. The reasons for exclusion were: age younger than 20 years (n = 1), too uncooperative to undergo the MR examination (n = 1), treatment with hyaluronic acid many years ago (n = 2), abnormal medial collateral ligament on routine MR images (n = 1), and medial meniscal tear on routine MR images (n = 1). Thus MR T2 values of unilateral knees were determined in 60 subjects divided into three age groups each containing 10 women and 10 men (see Table 1).

**Table 1:** Subject characteristics.

Subjects and Characteristics	Group 1	Group 2	Group 3
All subjects			
No. of subjects	20	20	20
Age (y)*	26.5±2.7	39.9±4.1	61.4±6.2
Body mass index* †	21.5±3.1	22.3±2.5	25.0±2.9
WOMAC pain score	0	0	0
Women			
No. of subjects	10	10	10
Age (y)*	25.8±1.8	39.7±3.9	61.4±5.3
Age range (y)	22–28	35–47	50–70
Body mass index*	20.2±2.7	21.3±2.3	25.9±2.9
Men			
No. of subjects	10	10	10
Age (y)*	27.2±3.4	40.0±4.6	59.7±5.9
Age range (y)	22–32	35–48	50–69
Body mass index*	22.7±2.9	23.3±2.5	23.8±2.8

* Mean values ± standard deviations.

† There was a significant difference (*P*<0.05) in BMI between Group 2 and Group 3 but not a significant difference in age between Group 1 and Group 2.

### Routine MR Imaging

All subjects were asked to rest one hour at least before the MR examination. Left knee menisci were selected as regions of interest (ROIs) only when the subjects' right knee did not fulfill the inclusion criteria; thus 53 right knees and 7 left knees were selected. The initial routine MR examination of the tibiofemoral joint involved taking proton density-weighted, T2-weighted, and fat suppression T2-weighted images in the coronal and sagittal planes. The MR images were interpreted by an experienced musculoskeletal radiologist (G.S.H, 21 years). All subjects in group 1 (age 20–34) had no MR evidence of cartilage lesions, while eleven volunteers (two men and nine women) had cartilage lesions in groups 2 (age 35–49) and 3 (age 50–70). Size of cartilage lesions (area of cartilage loss) was estimated to be a % of the cartilage surface area of each individual region and given a modified MRI Osteoarthritis Knee Score (MOAKS) [24] as follows: Grade 0 = none, grade 1<10%, grade 2 = 10–75%, and grade 3>75%. The femoral and tibial surfaces were divided into five separate regions: medial and lateral central femur (MFc and LFc), medial and lateral posterior femur (MFp and LFp), and medial and lateral anterior, central, and posterior tibia (MTa, MTc, and MTp; LTa, LTc, and LTp). Thus, the regions of the medial femorotibial joint (MFTJ) were the MFc, MFp, MTa, MTc and MTp and those of the lateral femorotibial joint (LFTJ) were the LFc, LFp, LTa, LTc, and LTp. The maximum MOAKS for the MFTJ and LFTJ was 30.

### MR Image Data Acquisition

All subjects were scanned in a supine position using a 3.0 T MR system (Achieva, Philips Medical Systems, Best, Netherlands). The selected joint of each subject was centered in an eight-channel knee coil (Philips Medical Systems). To minimize the magic angle effect on the meniscal T2 measurement [25], [26], the imaged leg was positioned straight with the long axis parallel to the main magnetic field (B_0_), and immobilized with MR-compatible plastic pads.

After obtaining pilot images in three orthogonal planes, 20 contiguous axial T1-weighted images were acquired using a spin-echo sequence for positioning with the following parameters: repetition time (TR) = 600 ms, echo time (TE) = 14 ms, number of excitations (NEX) = 1, matrix size = 256×256, slice thickness = 5 mm, and acquisition time = 2 min 35 s. Subsequently, oblique sagittal T2-weighted fat suppression images were obtained by using a multislice turbo spin-echo sequence. The sequence was prescribed to cover the medial and lateral menisci and had the following parameters: TR = 2500 ms; TE = 6.4, 9.4, 12, and 15 ms; echo train length = 3; slice thickness = 1 mm; slice gap = 1 mm; matrix size = 258×324 (zero-filled to 560×560); NEX = 2; 18 slices (9 slices per meniscus); and acquisition time = 14 min 40 s. Fat suppression was used to improve the tissue contrast of the images so that the derived T2 value was less prone to inaccuracy due to fat signal contamination from partial volume effects. One set of image data was discarded from subsequent statistical analysis because of poor image quality caused by severe motion artifact.

### Image Processing

#### Motion correction

After imaging acquisition was completed, all data were transferred to a stand-alone personal computer. A two-dimensional auto-correlation method was used for motion correction, in which the first image acquired with TE = 6.4 ms was used as a reference image to coregister the other three echo images acquired with TE = 9.4, 12, 15 ms, and between-slice two-dimensional correlation coefficients were calculated [16]. Since the signal intensities of all tissues except the menisci were similar at these TEs, the slices corresponding to the maximum correlation coefficient were regarded as having optimal coregistration. In this manner, all images were coregistered with the first-echo image (with minimal translational in-plane motion).

#### Region of Interest (ROI) definition

**Figure 2:**
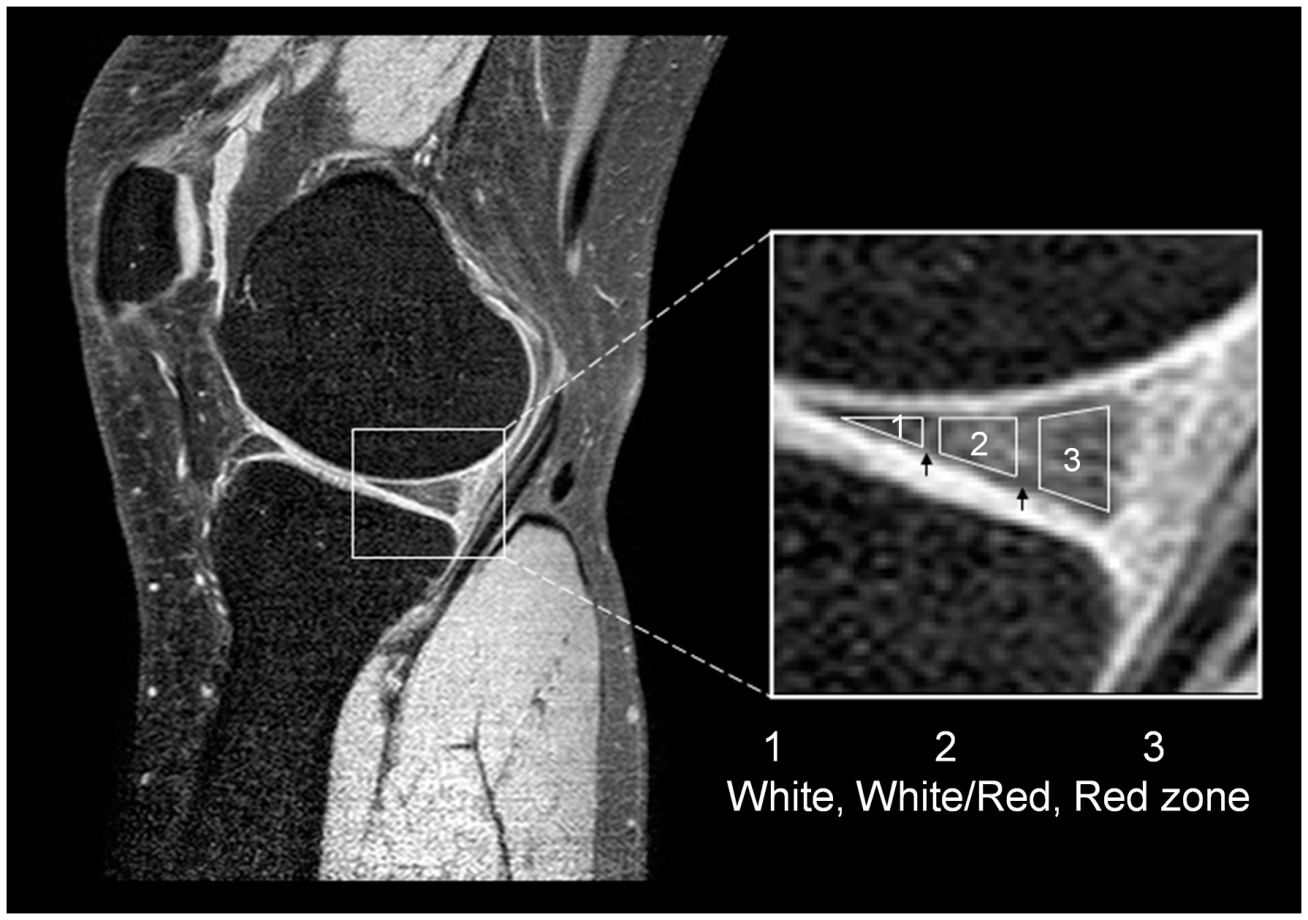
Representative ROIs in a nearly sagittal slice of a knee meniscus are shown. The selection of ROIs was performed on the first-echo MR image, which is enlarged on the right side. The black arrows indicate the separation between the three zones, and each zone was about one-third the width of the meniscus.

The ROIs (including the entire meniscus and three different meniscal zones) for measurements of the T2 values in the posterior horns of the medial and lateral menisci were identified on the sagittal MR images of the first echo (Figure 2). The three different meniscal zones have been previously described in detail [16]. The meniscus was divided on the basis of vascularization into the red zone (i.e., the outer zone or outer one-third of the meniscus), which receives a full blood supply [27], the white zone (i.e., the inner zone or inner one-third of the meniscus, which includes mainly fibrocartilage and is completely avascular [27]), and the red/white (R/W) zone (i.e., the middle zone or transition between the outer vascular and inner avascular zones [27]). To avoid partial volume effects, the upper and lower borders of the meniscus were not included in the ROIs. Two experienced operators (SWC and PHT, 2 years and 4 years) selected ROIs for three randomly selected subjects separately, discussed their disagreements together, and reached a consensus on the ROI selection procedure stated above to minimize discrepancies. The inter-operator disagreement in the ROI selection was less than 6%, leading to less than 4% discrepancies in the resulting T2 estimation for all subjects.

#### T2 calculation

To improve data fitting precision in the presence of noise and to minimize partial volume effect, analysis of the meniscal T2 values was conducted on a zone-by-zone basis. The mean signal intensities were calculated first in the posterior horns of the medial and lateral menisci on each of the motion-corrected images. Then, the T2 values were derived using the least square single-exponential curve-fitting method on the MATLAB 7.0 software platform (Mathworks, Natick, MA, USA). Goodness of fit was assessed by R^2^ values as used in nonlinear curve fitting [28]. The T2 values derived from each single image were then averaged over all slices to obtain mean T2 values and standard deviations for the entire red zone, white zone, and R/W zone in the posterior horns. In addition, averaged T2 values for the entire posterior horns of the meniscus were also obtained.

### Statistical Analysis

Paired-*t* tests with Bonferroni correction were used to compare the differences in T2 values between the medial and lateral menisci for the total region and the three different zones. Zonal dependence of T2 on age, BMI, and gender was analyzed using multiple linear regression with generalized estimating equations (GEE) and an exchangeable working correlation matrix to characterize the possible homogeneous correlations between T2 values. The GEE method with second-order age effect was used to fit the regression lines for the relationship between T2 values in the total posterior horns and participant age. Statistical analyses were performed on a personal computer using SPSS v.18.0 (SPSS, Chicago, IL). Statistical significance was defined as *P*<0.05.

## Results

The characteristics of all subjects are summarized in Table 1. The T2 values in the posterior horns of both menisci (lateral and medial menisci combined), medial and lateral menisci separately are summarized in Table 2. The T2 values in the posterior horns of both menisci for three zones are summarized in Table 3. Good curve fitting of the T2 data was obtained by using the nonlinear least-square algorithm (R^2^ = 0.998±0.058).

**Table 2:** Meniscal T2 measurements in different groups.

Subjects and menisci	T2 (msec)		
	Group 1	Group 2	Group 3
Women			
Both menisci	9.938±0.940	10.728±1.554	12.355±2.274
Medial meniscus	10.006±1.207	10.808±2.449	14.133±2.185
Lateral meniscus	9.863±0.501	10.628±1.090	10.800±2.265
Men			
Both menisci	9.169±0.744	9.644±0.671	10.945±1.330
Medial meniscus	9.191±0.744	9.732±0.559	11.338±0.752
Lateral meniscus	9.129±0.412	9.557±0.789	10.591±1.205

Note.–Data are mean values ± standard deviations.

**Table 3:** Meniscal T2 measurements in three zones.

Subjects	T2 (msec)		
	Group 1	Group 2	Group 3
Women			
White zone	8.019±0.608	9.086±1.356	11.183±1.885
R/W zone	9.243±0.945	10.163±1.583	12.293±2.642
Red zone	13.04±1.407	12.696±1.990	14.200±2.274
Men			
White zone	8.077±0.626	8.267±0.796	9.791±1.101
R/W zone	8.362±0.952	9.260±0.655	10.144±1.054
Red zone	11.497±1.283	11.237±1.123	12.396±1.839

Abbreviation: R/W zone, red/white or intermediate zone.

Note.–Data are mean values ± standard deviations.

### Age-dependent Variation in Posterior Horn T2 Values

**Figure 3:**
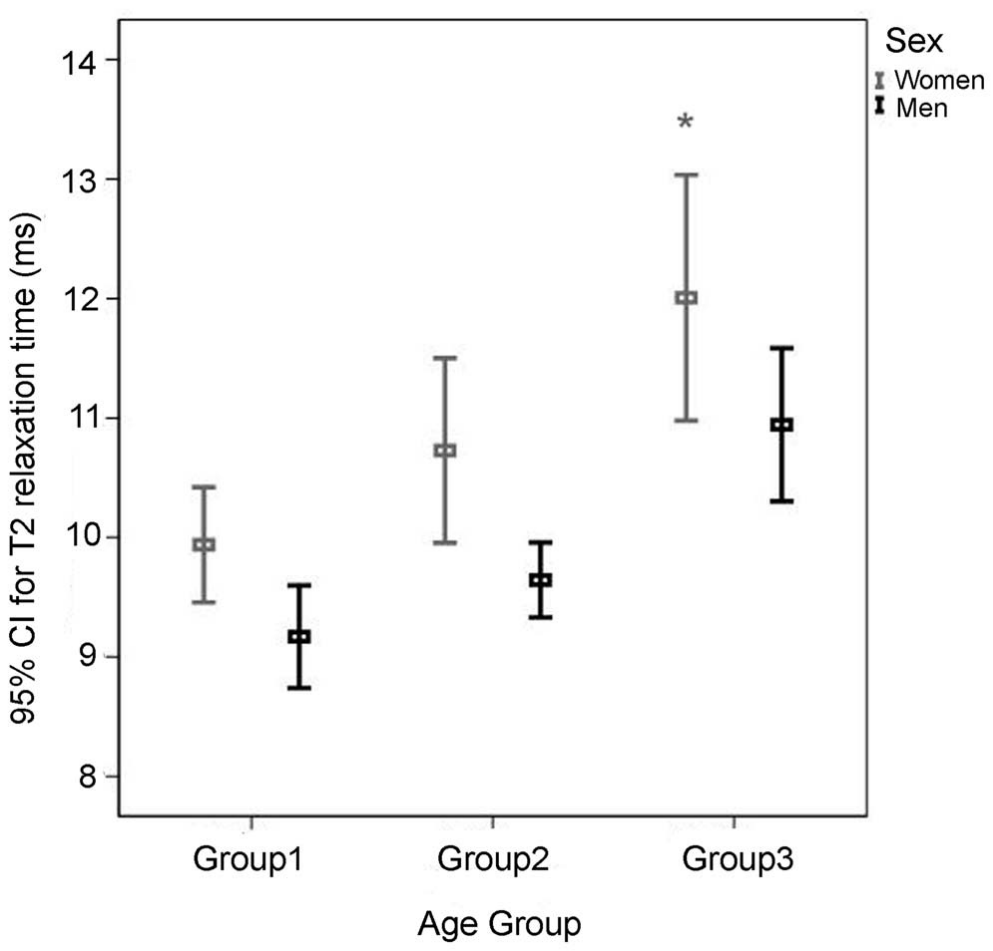
T2 values of both menisci for three age groups of women and men. In women, T2 values increase significantly from group 1 to group 3 (*P<0.001 as compared with group 1). Women show significantly greater T2 values than men in all three age groups (with *P* = 0.001, 0.004, and 0.049, respectively).

The results of multiple linear regression with GEE showed that both age and gender (with or without adjustment for the BMI effect) but not BMI (with adjustment for age and gender effects; *P* = 0.209; Table 4) significantly affected the T2 value in both menisci. More specifically, the T2 value (both menisci) was significantly higher in women age 50–70 and 35–49 (1.777 and 0.741 ms, respectively) than in women age 20–34 (*P*<0.001 and = 0.088, respectively; Table 4). The differences in T2 value (both menisci) among the three age groups were non-significant in men. Moreover, the differences in T2 value (both menisci) between the male 35–49 group and male 20–34 group and between the male 50–70 group and 20–34 group (0.313 and 0.109 ms, respectively) were lower (though not significantly) than these differences between the corresponding female age groups (*P* = 0.535 and 0.841; Table 4 and Figure 3). In addition, the T2 value (both menisci) was 0.878 ms lower in men than in women of the 20–34 group (*P* = 0.001; Table 4), and 1.084 and 1.062 ms lower in men than in women (*P* = 0.004 and = 0.049, respectively) of the 50–70 and 35–49 groups.

**Table 4:** Comparisons of meniscal T2 values among three age groups and two gender groups after adjusting for the BMI effect in multiple linear regression analysis with GEE.

			95%		Wald	
Parameters	B	SE	Confidence	Interval	Chi-square	*P*-value
BMI	0.052	0.042	–0.029	0.134	1.578	0.209
Sex (M vs. W)	–0.878	0.266	–1.399	–0.358	10.926	0.001
Group 3 vs. Group 1*	1.777	0.473	0.850	2.704	14.117	<0.001
Group 2 vs. Group 1*	0.741	0.434	–0.109	1.591	2.920	0.088
Sex (M)×Group 3	–0.109	0.544	–1.176	0.958	0.040	0.841
Sex (M)×Group 2	–0.313	0.504	–1.301	0.676	0.385	0.535

* Group 1∶ 20
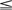
Age
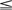
34; Group 2∶ 35
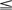
Age
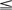
49; Group 3∶ 50
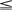
Age
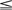
70 (Group 1 is the reference group).

Sex (M: Men; W: Women [the reference group]).

Abbreviations: BMI, body mass index; B, regression coefficient; SE, standard error.

### Location-dependent Variation in T2 Value

**Figure 4:**
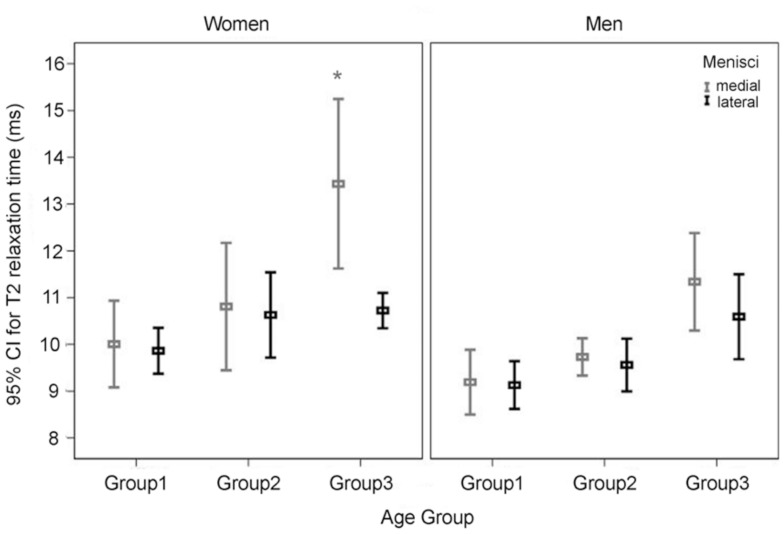
T2 values of the medial and lateral menisci in three age groups of women and men. The T2 values of the medial meniscus for women increase significantly from group 1 to group 3 (*P<0.001 as compared with group 1). But the T2 values of the lateral meniscus for women and men were not significantly different among three age groups.

Analysis of the T2 values of the medial and lateral menisci using the multiple linear regression model (Table 5) and after adjusting for the effects of age and gender, showed no significant effect of BMI on T2 values in the medial and lateral menisci (*P* = 0.606 and 0.239, respectively) of subjects with BMI <30 kg/cm^2^. Gender also had no effect on T2 values in medial and lateral menisci for subjects aged 20–34 (*P* = 0.186 and 0.085, respectively). However, the T2 value in the medial menisci was significantly higher for women age 50–70 than for women aged 20–34 years (*P*<0.001) but not for women aged 35–49 (*P = *0.251). Although significant in women, the differences in the T2 value in the medial menisci among the three age groups were non-significant in men. On the other hand, differences in the T2 value in the lateral menisci among the three age groups were non-significant in both men and women (Table 5 and Figure 4). Medial menisci were found to contribute significantly to the age-related variation in T2 values of both women and men (Figure 4). T2 value was significantly higher in the medial menisci than those in the lateral menisci in only women age 50–70 (*P* = 0.006). Medial-lateral differences for all three zones were not significant in women and men in all three age groups.

**Table 5:** Comparisons of T2 values of medial/lateral menisci among three age groups and two gender groups after adjusting for the BMI effect in multiple linear regression analysis.

			95%		Wald	
Parameters	B	SE	Confidence	Interval	Chi-square	*P*-value
**Medial**						
BMI	0.038	0.074	–0.107	0.184	0.266	0.606
Sex (M vs. W)	–0.929	0.703	–2.307	0.449	1.745	0.186
Group 3 vs. Group 1*	3.190	0.812	1.599	4.781	15.451	<0.001
Group 2 vs. Group 1*	0.754	0.657	–0.534	2.042	1.317	0.251
Sex (M)×Group 3	–1.093	1.012	–3.077	0.891	1.166	0.280
Sex (M)×Group 2	–0.224	0.923	–2.032	1.585	0.059	0.808
**Lateral**						
BMI	0.052	0.044	–0.034	0.138	1.388	0.239
Sex (M vs. W)	–0.795	0.462	–1.702	0.111	2.960	0.085
Group 3 vs. Group 1*	0.570	0.454	–0.320	1.461	1.575	0.209
Group 2 vs. Group 1*	0.699	0.407	–0.099	1.496	2.950	0.086
Sex (M)×Group 3	0.756	0.598	–0.416	1.929	1.598	0.206
Sex (M)×Group 2	–0.364	0.598	–1.536	0.808	0.371	0.543

* Group 1∶ 20
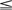
Age
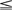
34; Group 2∶ 35
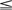
Age
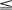
49; Group 3∶ 50
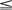
Age
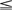
70 (Group 1 is the reference group).

Sex (M: Men; F: Women [the reference group]).

Abbreviations: BMI, body mass index; B, regression coefficient; SE, standard error.

### Zonal-dependent Variation in T2 Value

**Figure 5:**
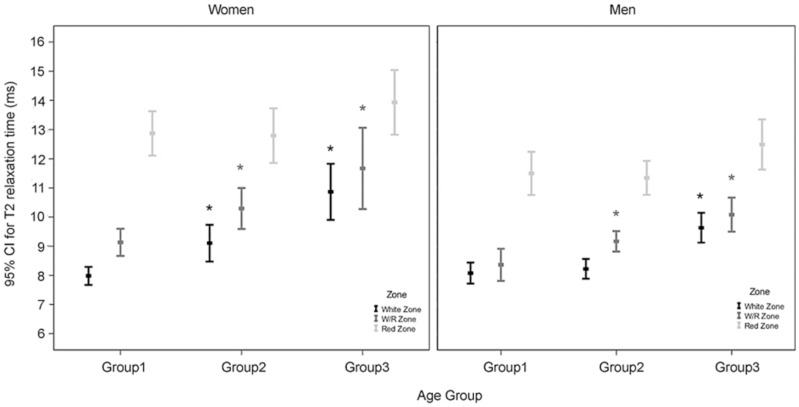
T2 values of the three zones in three age groups of women and men. The differences in T2 values for the white zone and R/W zone in women and men are significantly greater between group 3 and group 1 and between group 2 and group 1 (*P<0.01) but no corresponding differences exist for the red zone in women and men.

Multiple linear regression analysis after adjusting for the effects of age and gender showed that BMI had no significant effect (*P* = 0.186) on zonal T2 values (not shown). Table 6 showed the multiple linear regression analysis of differences in zonal T2 values among three age groups. More specifically, the T2 values in the white zone were significantly higher for women aged 50–70 and 35–49 than for women aged 20–34 (2.470 and 1.024, ms respectively; *P*<0.001 and = 0.009, respectively). R/W zone (*P*<0.001 and = 0.004, respectively) T2 values but not red zone (*P* = 0.071 and 0.905, respectively) T2 values were significantly higher in women of all three age groups. Moreover, the age-related differences in white zone T2 values were significantly lower in men than in women (–1.019 and –0.962 ms; both *P* = 0.035; Table 6 and Figure 5). The white zone and R/W zone were found to contribute significantly to the age-related variation in T2 values of both women and men (Figure 5). On the other hand, for both men and women in all age groups, T2 values tended to increase from the white zone, R/W zone, to the red zone (Table 3 and Figure 5).

**Table 6:** Comparisons of zonal T2 values among three age groups and two gender groups after adjusting for the BMI effect in multiple linear regression analysis with GEE.

			95%		Wald	
Parameters	B	SE	Confidence	Interval	Chi-square	*P*-value
**White zone**						
Group 3 vs. Group 1*	2.496	0.4369	1.640	3.353	32.636	<0.001
Group 2 vs. Group 1*	1.071	0.3916	0.303	1.838	7.475	0.006
Sex (M)×Group 3	–1.019	0.4834	–1.967	–0.072	4.447	0.035
Sex (M)×Group 2	–0.962	0.4571	–1.857	–0.066	4.424	0.035
**Red/White zone**						
Group 3 vs. Group 1*	2.767	0.5556	1.678	3.856	24.803	<0.001
Group 2 vs. Group 1*	1.161	0.4033	0.370	1.951	8.282	0.004
Sex (M)×Group 3	–1.059	0.6360	–2.305	0.188	2.771	0.096
Sex (M)×Group 2	–0.357	0.4585	–1.255	0.542	0.605	0.437
**Red zone**						
Group 3 vs. Group 1*	1.171	0.6485	–0.100	2.442	3.260	0.071
Group 2 vs. Group 1*	–0.079	0.6600	–1.373	1.214	0.014	0.905
Sex (M)×Group 3	–0.181	0.7847	–1.718	1.357	0.053	0.818
Sex (M)×Group 2	–0.077	0.7615	–1.569	1.416	0.010	0.920

* Group 1∶ 20
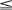
Age
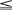
34; Group 2∶ 35
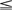
Age
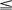
49; Group 3∶ 50
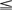
Age
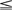
70 (Group 1 is the reference group).

Sex (M: Men; F: Women [the reference group]).

Abbreviations: BMI, body mass index; B, regression coefficient; SE, standard error.

Eleven volunteers had degeneration of the tibiofemoral joint cartilage: two were women aged 35–49 with MOAKS score 3; two, men aged 50–70 with score 4; four, women aged 50–70 with score 4; and three, women aged 50–70 with score 6. Pearson correlation coefficient analysis failed to find any significant correlation between the T2 value (both menisci) and MOAKS score (R = 0.389, *P* = 0.132).

## Discussion

In this study, the age-related increase in T2 values was more prominent in the medial meniscus than that the lateral meniscus. In women age 50–70, T2 was significantly higher in the medial meniscus posterior horn than in the lateral meniscus posterior horn. This location-dependent variation may be attributed to the presence of less collagen, less proteoglycans, and more water in the medial meniscus than the lateral meniscus [29], [30]. Under normal conditions, proteoglycans are responsible for hydration within the meniscus, restricting water movement and adding compressive stiffness to the meniscus. The scarcity of proteoglycan in the medial menisci leads to a concomitant increase in the space inside the porous matrix, which in turn is filled by an influx of water in a way similar to that which occurs in the OA patients [11] and in the articular cartilage [9]. As the mobility and the amount of water both increase, the regional T2 value increases [11], [12], suggesting susceptibility to degenerative damage is elevated in the medial menisci. Previous reports demonstrated a higher prevalence of meniscal abnormalities in the posterior horn of the medial meniscus in OA patients [11], [12], which is in agreement with the location-dependent T2 variations in our study.

Due to zonal differences in meniscal composition and vascularization [18], T2 value increased steadily from the white zone to the red zone in all age groups, which confirms initial observations made in young populations [16]. In addition, the age-related variation in T2 also depended on zone, being significantly greater in the white and R/W zones than in the red zone, especially in women. In fact, during the natural degeneration of human meniscus, one of the most common degenerative tears (the horizontal-cleavage tear) usually begins near the inner margin of the meniscus and extends out toward the periphery [31], [32], suggesting that the white zone may be more prone to early degenerative tears than the R/W and red zones. This is consistent with our observation of a higher rate of T2 value increase in the white zone. Moreover, since the zonal dependence of meniscal healing has been reported in patients undergoing anterior cruciate ligament reconstruction [33], further investigations of zonal differences in meniscal T2 may identify possible potential factors for assessing meniscal healing [34], [35]. All of these studies reconfirm the value of regional analysis.

OA is more prevalent in women [36]. Several studies evaluated the effect of gender on meniscal morphology and biomechanics [35], [37], [38]. Kerrigan et al. indicated that a gender difference in walking pattern could result in a difference in mechanical stress and could thereby account for the difference in OA prevalence [39]. Webber et al. examining gender differences in meniscal fibrochondrocyte quantity and proliferation showed that their number was greater in women than in men, regardless of age [40]. Although the mechanism is complicated and controversial, our current results demonstrate that T2 values in the posterior meniscal horns are higher in women than in men regardless of age, suggesting the need for investigation of this gender difference during meniscal degeneration.

To our knowledge, this is the first study to investigate age-, gender-, location-, and zone-related changes in the meniscus using noninvasive MR T2 values. T2 values of both posterior meniscal horns were found to increase with increasing age, possibly reflecting disruption of the articular cartilage architecture and consequent changes in water content [9], [41], [42]. Furthermore, previous papers indicated that marked collagen content decreases are accompanied by exponentially increased pentosidine concentrations and increased water content during degeneration of the menisci and lead to loss of elasticity, less solubility of collagen, and less digestibility of collagen [15], [18], [43]. Consequently, information about the effect of age on meniscal degradation and the initiation of OA can be non-invasively obtained from an assessment of change in T2 value. Rauscher et al. mentioned that gross analysis of the menisci without investigation of zonal variation provides only limited information [14]. In our study, analysis of zonal characteristics disclosed the age-related differences in MR T2 values.

In a previous study comparing healthy subjects and patients with OA, meniscal T2 values were significantly correlated with cartilage WORMS score [11]. Although, in our study, the correlation between meniscal T2 values and cartilage MOAKS score was not significant, a trend toward correlation was noted, with correlation coefficient R^2^ value similar to that found in the previous study. The point of difference between the previous study and our study may be the cartilage, which in our subjects was not severely damaged by OA.

Our study had several limitations. First, we placed the ROIs manually for T2 estimation. Drawing the ROIs manually could be prone to operator-dependent error. Thus we assessed the inter-operator agreement before assessing the image data of our 60 subjects. The low interoperator variability of less than 4% in T2 seemed to be minor compared with the zonal/gender/location/age differences. Second, the signal intensity and T2 value may vary because of the magic angle effect which depends on the orientation of the cartilage fiber relative to the main magnetic field [25]. In this study, the magic angle effect was nullified by not bending the knee and always positioning the long axis of the leg parallel to the main magnetic field. Nevertheless, some of the T2 deviations observed in our study may still be due to changes in cartilage fiber orientation. Third, after grouping according to age and gender, each group had only ten subjects. While studies with small numbers of subjects are prone to selection bias, in this prospective study we used wide inclusion criteria so that a relatively large number of asymptomatic subjects could be recruited. The statistical power of our data seemed to be sufficient to preliminarily identify factors that may affect meniscal T2. Therefore, findings from our study should at least guide hypothesis generation and thereby assist larger scale investigations in the future. Fourth, we investigated only the posterior meniscal horn because it is highly susceptible to injury and degradation based on its anatomic and biomechanical architecture [29], [41]. It will be crucial in a more advanced study to comprehensively investigate the MR T2 behavior in different segments of the knee meniscus for early detection and clinical management of degeneration of the meniscus, including the body and the anterior horn. Finally, T2 measurement in the meniscus is only an indirect index reflecting possible disruption of cartilage architecture and consequent changes in water content. Even if validation of these changes histologically and biochemically [11] adds diagnostic value to meniscal T2 measurement, the specificity of meniscal T2 value prolongation remains to be explored in more comprehensive examinations.

### Conclusion

The MR T2 values of the posterior meniscal horns are diverse and related to age, gender, location, and zone. The T2 value findings in this study may increase our understanding of meniscal disease.
